# Mind Reading

**Published:** 1887-08

**Authors:** 


### HALL'S JOURNAL OF HEALTH

TRUTH DEMANDS NO SACRIFICE; ERROR CAN MAKE NONE.

VOL. 34. AUGUST, 1887. No. 8.

### MIND READING.

Mind reading, or thought transference, as some people prefer to call it, is only a single element of a sublime truth. It belongs to a class of phenomena wholly allied to our spiritual being, which is only just beginning to be recognized and scientifically investigated. Even the London Psychological Society, with all its conservatism, has reached a point of confessed belief in this faculty of the human brain, yet it is only the verge of an almost unexplored sea. For centuries past, the votaries of science have confined their researches almost exclusively to *physics*, in which field great and wonderful discoveries have been made of vast advantage to mankind. Many of the settled opinions of the past concerning the processes of nature and the commonest elements which support life, have been shown to have been founded in error, and a clearer perception of the truth has been given; until now, the classes of our public schools are better and wiser taught than were the most pretentious schoolmen a few centuries ago. But while the names of those who have led the way in the various departments of learning are entitled to all honor, there are others whose researches into the subtler and more intricate faculties of the soul are no less praiseworthy, who have found it hard to gain even a becoming recognition, to say nothing of the world's applause.

It is only a few months since Mr. W. Irving Bishop created a wide spread sensation in Boston by exhibiting his faculty of mind reading, or the receptivity of his own mind, to receive impressions from the minds of others, in the presence of certain local notabilties, who were present by his invitation, among whom were the well known clergyman Minot J. Savage and the Mayor of the city. One feature of the exhibition consisted in hiding a little gold scarf pin several blocks distant from the exhibition hall, under some kindlings, within an open fire grate in an upper room of a private residence. With the hiders of the pin, Mr. Bishop entered a carriage, blindfolded, and taking the reins in hand, drove rapidly in the direction of the hiding place. Alighting some distance from it, he made sure his way along the side-walk, and, entering the house, ran up stairs, passed into the front room and began fumbling among the before-mentioned kindlings. In doing this, he quite accidentally drew some waste paper over the pin, so that Mr. Savage, who was present, could not see it. The effect of this upon Mr. Bishop was quite extraordinary, for, notwithstanding, he still clung to the idea that the *pin* was somewhere about the grate, his search was so much at random, that, after some minutes, it was temporarily abandoned. Then Mr. Bishop was led from the room, the pin once more placed where Mr. Savage could see it, and Mr. B. upon being again led in, still blind-folded, had little difficulty in finding it. This circumstance would seem to demonstrate that in experiments with Mr. Bishop as a sensitive, the transmitter of thought must not only steadfastly retain a mental picture of the object hidden, but he must also have a *visual* perception of it.

Some writers contend that mind reading has, in reality, nothing to do with the result of these experiments ; that it all depends upon the involuntary action of the muscles, or, more definitely, that the mind, conscious of the hiding place of an object, is in a continual state of nervous action during the search, thereby causing the muscles to contract or relax in such manner as to indicate, by contact, the right direction, but this theory, for it is nothing more, has been too often disproved to leave it any standing place. We have known experiments in thought transference to be successfully performed by way of amusement, at social gatherings, the immediate participants being novices who had no previous knowledge of their capacity in that direction, and were themselves among the more astonished at the result.

It must be borne in mind, too, that many and various other evidences have been had of mind transference without personal contact, equally, if not more extraordinary than the foregoing, which can only be accounted for upon the hypothesis of an intimate psychological agreement between individual minds for the time being, reciprocally acting and being acted upon in the transmission of thought.

Mr. Bishop does not pretend to account for these phenomena. A number of years ago, in the course of his exhibitions, he was accustomed to refer to the spiritual theory and those who upheld it, in very disparaging terms, but more recently, we have heard nothing of this sort of disparagement, although he endeavors still to curry favor with the conservative element, from which he derives the greater share of his patronage. This, as an evidence of his business tact, is almost equal to his sensational methods of advertising himself and his little hobby. That he is, in fact, a sensitive or psychic, there can be no doubt. Indeed, there is no other way of accounting for the manifestations of which he is the instrument. Had he been sufficiently patient and modest to allow his psychologic gifts to develope and strengthen by intelligent use and direction, he might, and probably would, have entered a much broader field of usefulness, than the one which he occupies for the present, if less profitable, as it is almost sure to have been.

In writing upon these matters, Dr. J. R. Buchanan says of them and of Mr. Bishop :

" The performances of Mr. Bishop at Music Hall, were successful and remarkable in demonstrating clairvoyance and thought reading. Their success was universally admitted, and there the leaders of Boston skepticism—Dr. Holmes, Col. Higginson and a host of similar skeptics, were compelled to recognize these facts ; and the daily press records the triumph of Bishop without a word of dissent or criticism.

But, then, 'what will they do about it?' Will the gentlemen who have so long denied and derided eternal truths and ostracised the humbler and wiser students of nature, who have accepted them, make any amends for past injustice? Will there be any apology to those who have been robbed of reputation and position, or any tardy honor accorded to the veterans of progress? Probably not. Harvey was never recompensed for the injustice of his opponents. To the last moment he felt the sting of ingratitude. They who begin by being unfair and unjust, cannot be expected to set an example of generosity or justice. A few, a very few, of the skeptical class, will be honest enough to frankly recognize the truth. Rev. M. J. Savage frankly declares in the *Herald* that he considers mesmerism of great therapeutic value, and considers clairvoyance and thought transference even to immense distances, established beyond doubt. But when will the false philosophies of the universities be replaced by true science? When will the citadels of hereditary ignorance be captured ?

What bearing has all this upon Spiritualism? Mr. Bishop shuns that subject and seeks the favor of orthodoxy. He denies any spiritual participation in his performances. But he is no philosopher, and his assertion is of no importance when we know that he has done things heretofore, which cannot be done without spiritual co-operation. Nor can any one manifest his high psychic powers without being accessible to spiritual influences.

Mr. B. has done well in coming before the Boston aristocracy as a simple exhibitor without a theory. If he had given Spiritualism any credit, they would have been repelled. If he had professed to teach the philosophy of what he exhibited, he would have offended the self-love of Boston, which does not tolerate any wisdom superior to its own. As a mere exhibitor, he disarmed criticism, and the *elite* of the city flocked to his exhibitions. After this, skepticism may possibly shun the subject, but will hardly have the hardihood to deny what Bishop has proved. From such experiments, it is a short step to the spiritual philosophy."

We venture to say there are hundreds, nay, thousands of persons living all about us, who possess this faculty of *mind reading* in a degree approaching Mr. Bishop, who have no knowledge of it themselves, and which, if encouraged and given room to expand by cultivation, would develop higher phases of *psychism* for example, *clarivoyance*, which is an independent faculty, so far, at least, as other minds are concerned.

That Mr. Bishop has never approached this is made clear by the circumstance that, in all his experiments, it is indispensable that the mind concerted with his own should be intently fixed upon the object, or that feature of it which he is to divulge, for example, the hands of a watch denoting the time, or the number of a bank note. Without this correspondence, he is bewildered and powerless of results, as evidenced in the case of the concealed pin: the moment it was lost to the view of his attendant, the current was broken, so to speak, and no progress could be made till the circuit was restored. The clairvoyant needs no such correspondencies. In a trance state, or otherwise, according to his habit, he is able to describe things and occurrences at long distances, which no mind present comprehends and no physical eye is able to see. As compared with this, Mr. Bishop's field is the *kindergarten*. But it is, perhaps, as large a dose as our orthodox brethren are capable of receiving, and, at the same time, reconciling with their opposition to the *spiritual* theory, to which all earnest investigators are driven who pass beyond the plane of amusement into that of sober inquiry.

We would not highly esteem an explorer who would voluntarily forsake the main channel of a great water way for one of its smaller tributaries, lest further investigation should compel him to make essential changes in his map already published ; yet such a course fairly illustrates the position of a very numerous class, who shun the truths which have been brought to light concerning matters of far weightier consequence to them and to the world at large. A mind obstinately committed to error is akin to a mind diseased, and almost as valueless to mankind.

### LACTATED FOOD.

Wells, Richardson & Co.'s Lactated Food is gaining in favor every day. Those who have fully tested its qualities are loud in its praise, This is the season of its greatest demand for nursery use.

